# Taking Advantage of the Senescence-Promoting Effect of Olaparib after X-ray and Proton Irradiation Using the Senolytic Drug, ABT-263

**DOI:** 10.3390/cancers14061460

**Published:** 2022-03-12

**Authors:** Camille Huart, Maude Fransolet, Catherine Demazy, Benjamin Le Calvé, Stéphane Lucas, Carine Michiels, Anne-Catherine Wéra

**Affiliations:** 1 Cellular Biology Research Unit (URBC), Namur Research Institute for Life Sciences (NARILIS), University of Namur (UNamur), 5000 Namur, Belgium; camille.huart@unamur.be (C.H.); maude.fransolet@gmail.com (M.F.); catherine.demazy@unamur.be (C.D.); benjamin.lecalve@gmail.com (B.L.C.); carine.michiels@unamur.be (C.M.); 2 Laboratory of Analysis by Nuclear Reaction (LARN), Namur Research Institute for Life Sciences (NARILIS), University of Namur (UNamur), 5000 Namur, Belgium; stephane.lucas@unamur.be; 3 Molecular Imaging, Radiation and Oncology (MIRO) Lab, Institut de Recherche Expérimentale et Clinique (IREC), Université Catholique de Louvain (UCLouvain), 1200 Woluwe-Saint-Lambert, Belgium

**Keywords:** cancer, PARP, proton radiation, X-ray radiation, senescence, senolytics

## Abstract

**Simple Summary:**

Radiotherapy is one of the most common treatments for cancer. Overcoming the failure and side effects of radiotherapy are current challenges. It has been recently demonstrated that senescence contributes to radioresistance. Cellular senescence is a permanent arrest in cell proliferation induced by various factors, such as radiation. Here, we aimed to assess the potential of combining the radiation and DNA damage repair inhibitor, Olaparib to a senolytic drug, ABT-263. We demonstrated that combining radiation, Olaparib and ABT-263 successfully targeted the radio-induced senescent cells resulting in increased cell death and reduced senescence-associated secretory phenotype. These results paved the way towards a new therapeutic combination for patients treated with radiotherapy and Olaparib.

**Abstract:**

Radiotherapy (RT) is a key component of cancer treatment. Although improvements have been made over the years, radioresistance remains a challenge. For this reason, a better understanding of cell fates in response to RT could improve therapeutic options to enhance cell death and reduce adverse effects. Here, we showed that combining RT (photons and protons) to noncytotoxic concentration of PARP inhibitor, Olaparib, induced a cell line-dependent senescence-like phenotype. The senescent cells were characterized by morphological changes, an increase in p21 mRNA expression as well as an increase in senescence-associated β-galactosidase activity. We demonstrated that these senescent cells could be specifically targeted by Navitoclax (ABT-263), a Bcl-2 family inhibitor. This senolytic drug led to significant cell death when combined with RT and Olaparib, while limited cytotoxicity was observed when used alone. These results demonstrate that a combination of RT with PARP inhibition and senolytics could be a promising therapeutic approach for cancer patients.

## 1. Introduction

About 50% of cancer patients undergo radiotherapy during the course of their treatment either for curative prospect or palliative assistance [1]. In order to improve patient outcome, treatments usually combine surgery, chemotherapy and radiotherapy. Although improvements have been made over the last decades to optimize the therapeutic index, resistance to treatment is still a major issue. Amongst the strategies to optimize cell response to radiation, it is worth mentioning the improvement of radiation dose conformity, higher effectiveness radiation and biological strategies [2,3,4].

The improvement of dose spatial distribution aims at dose escalation, hypofractionation and the sparing of healthy tissues. For photons, techniques such as intensity modulated radiation therapy (IMRT) must be favored as well as stereotactic body radiotherapy (SBRT), which is being pushed forward. In the seeking of dose conformation, charged particles, such as protons or carbon ions, present a clear advantage compared to photons due to their depth dose profile [5,6]. While photons deposit most of their energy close to the surface entrance, followed by a continuous decrease characteristic in their attenuation, charged particles deposit a small fraction of their energy before what is called the Bragg peak. This peak characterizes the maximal energy released when the particles come to rest. For photons, dose deposition upstream and downstream the tumor is observed, but for charged particles, the dose sharply decreases beyond the Bragg peak, allowing the downstream tissues to be spared. Highly conformal dose deposition profiles can be obtained as the position of the Bragg peak can be tuned with the incident beam energy to coincide with the tumor position.

A wide range of biological strategies are under study as well as in clinical assessment for charged particles and conventional photon irradiation [7,8]. One of the most promising strategies is that of DNA repair inhibitors. Amongst them, the most widely used is poly(ADP-ribose)polymerase (PARP) inhibitor (PARPi) [9,10]. PARPi are able to stabilize the single strand breaks (SSBs) induced by radiation, which are then translated into double-strand breaks (DSBs) at the replication fork leading to increased cell death. This is particularly observed for homologous recombination (HR) deficient cell lines as these DSBs are usually processed with the HR machinery during the S phase of the cell cycle. This is generally referred to as synthetic lethality [11]. PARPi are currently approved by the FDA for patients with germline BRCA-mutated cancers, such as ovarian cancer, breast cancer, pancreatic cancer and prostate cancer [12,13,14,15]. Combination strategies with RT and/or chemotherapy might extend the therapeutic indications of PARPi to patients with BRCA wild-type tumors, as sensitization has been observed in several studies [16,17,18,19].

Although the literature is scarce for proton irradiation [19,20], several authors have demonstrated that treatment with radiotherapy and/or PARPi triggers cell senescence [21,22,23,24]. Senescence is marked by changes in cell morphology, irreversible cell cycle arrest characterized by an increase in p16^INK4A^ and p21^Waf1/Cip1^ expression, increased senescence-associated β-galactosidase (SA-β-gal) activity, changes in gene expression, Lamin B1 loss, presence of senescence-associated heterochromatin foci (SAHF) and a common mitochondrial DNA deletion of 4977 bp [25,26]. In addition, senescent cells are characterized by a specific senescence-associated secretory phenotype (SASP). SASP factors include chemokines, inflammatory cytokines, matrix-remodeling proteases, growth factors and associated proteins, as well as insoluble secreted proteins [27]. Senescent cells exhibit an increased resistance to apoptosis, thereby impacting tumor growth [28]. Chronic accumulation of senescent cells has been proposed to support SASP signaling as well as to promote pro-tumoral effects on the surrounding microenvironment [29]. The pro-tumoral ability of senescent cells was discovered when senescent fibroblasts were co-cultured with pre-neoplastic epithelial cells, which led to an increased proliferation rate of the epithelial cells. These pro-tumoral abilities have been, in part, linked to the secretion of SASP factors, notably IL6 and IL8 [30,31,32,33]. In addition, although senescence is often described as an irreversible state, several studies have reported proliferation in the recovery of cells from their induced senescence status. For example, Fleury et al. recently demonstrated the need for sustained PARPi therapy as drug withdrawal permitted senescent cells to escape the senescent state and to re-initiate proliferation [21]. Alotaibi and colleagues demonstrated in HCT-116 cells that Olaparib promoted senescence induced by radiation and that a sub-population of these cells was able to re-enter proliferation [24]. It is now suggested that senescence could represent a form of tumor dormancy [34]. Associated with its pro-tumoral secretory phenotype, this population could ultimately lead to treatment failure.

For this reason, the clearance of senescent cells has become an interesting new pharmacological strategy. These molecules which are able to kill senescent cells are called senolytics [35]. The goal of these drugs is to selectively eliminate senescent cells while sparing normal cells. Several companies are currently developing senolytics, exploiting the dependence of senescent cells for specific pro-survival pathways [36]. Examples are ABT-263 and TW-37, which inhibit pro-survival genes in the Bcl-2 family [37]. These drugs specifically induce apoptosis in RT- and PARP inhibitor-induced senescent cells [21,22,23].

In this work, we examined the effects of X-rays and protons combined with Olaparib on BRCA wild-type cancer cells. We showed that the combination of Olaparib with radiation (both photons and protons) increased the number of senescent cells, which can subsequently be targeted by ABT-263. Importantly, the concentration of Olaparib and ABT-263 used did not induce cellular senescence or cell death if not combined to radiation. While Olaparib radiosensitized all cell lines, the proportion of senescence induced when combined with radiation was cell line-dependent and the efficiency of the combined treatment with ABT-263 varied accordingly. Together, our results propose a new therapeutic approach combining protons or X-rays and Olaparib with senolytic agents to decrease the number of senescent cells, enhancing cell death and reducing the risk of treatment failure.

## 2. Materials and Methods

### 2.1. Cell Culture, Olaparib and ABT-263

Human A549 non-small-cell lung cancer (NSCLC) cells were sub-cultured in Glutamax Modified Eagle’s Medium (Gibco Life Technologies, Carlsbad, CA, USA) supplemented with 10% fetal bovine serum (FBS) (Gibco Life Technologies). KP4 pancreatic cancer cells and HCT-116 colon cancer cells were sub-cultured in 4500 mg/L glucose Dulbecco’s Modified Eagle’s Medium (Gibco Life Technologies) supplemented with 10% FBS. For the experiments, the medium was supplemented with 0.1% penicillin/streptomycin (Sigma, Saint Louis, MO, USA). Olaparib (AZD2281, S1060, Selleckchem, Houston, TX, USA), a poly(ADP-ribose)polymerase inhibitor, was used at 0.5 µM final concentration. ABT-263 (Navitoclax, MedChemExpress, HY-10087, MedChemExpress, Monmouth Junction, NJ, USA), a Bcl-2 family inhibitor, was used at 1 µM final concentration. Cells were incubated in the presence of the Olaparib for a total duration of 24 h. ABT-263 was added 24 h after irradiation and was left up to the end of the assay. The concentration chosen for Olaparib is based on previous work [19]. For ABT-263, 1 µM concentration allowed to limit the clonogenic toxicity when combined with Olaparib in cells unexposed to radiations.

### 2.2. Irradiation: Protons and X-rays

The experimental set-up and irradiation procedure for protons using a 2MV tandem accelerator are described in [38,39]. Briefly, 24 h before irradiation, A549 cells were seeded as a 38 µL drop (800 c/µL) in designed irradiation chambers and left to attach to a Mylar foil for 4 h. The chambers were then filled with medium and left in the incubator before the irradiation. A proton beam energy of 1.3 MeV was used for the experiments, which corresponds to a fixed linear energy transfer (LET) of 25 keV/µm within the cell monolayer. Such LET is found at the end of each particle’s track composing the proton beams found in clinic. This high LET leads to the highest effect of protons in cells [40]. Dose rates ranging from 2 to 8 Gy/min were used.

For X-ray irradiation, cells were seeded in 24 well-plates 24 h before irradiation to obtain the same density as for proton irradiation (200 × 10^3^, 250 × 10^3^ and 300 × 10^3^ cells/well for A549, KP4 and HCT-116, respectively). Irradiations were performed at 225 kV (X-RAD 225-XL, PXI) and the dose rate set to 2 Gy/min.

For proton and X-ray irradiations, 2 h before irradiation, the culture medium was replaced with medium containing: no inhibitor (CTL) or 0.5 µM Olaparib (Ola.). Within 30 min after the irradiation, cells were detached using trypsin, then counted and seeded in cell culture plates. After 24 h, the medium was refreshed with FBS-supplemented medium containing or not containing ABT-263 at 1 µM and cells were left to proliferate.

### 2.3. Senescence-Associated Beta-Galactosidase

Six days post-irradiation, cells were detached and around 15,000 cells were reseeded in 12 well-plates in duplicates. The next day, cells were fixed with 2% formaldehyde, 0.2% glutaraldehyde in PBS for 5 min. Cells were rinsed twice with PBS and incubated at 37 °C for 18 h (A549 cells) to 24 h (KP4 and HCT-116 cells) in the staining solution containing: x-gal (20 mg/mL) (No. 0428-1G, Amresco, Fountain Parkway Solon, OH, USA), phosphate buffer (pH 5.8), potassium ferricyanide (100 mM), potassium ferrocyanide (100 mM), NaCl (2.5 M) and MgCl_2_ (1 M). After incubation, cells were rinsed twice with PBS and twice with methanol before observation under optical microscope [41]. At least 200 cells were counted. At least three independent experiments were performed, and data are presented as mean ± 1 SD.

### 2.4. EdU Labelling

Six days after irradiation (5 Gy for A549 and KP4 cells and 3 Gy for HCT-116 cells due to a higher radiosensitivity), cells were detached and reseeded onto a glass cover-slip. The next day, cells were incubated for 8 h with 10 µM EdU before 4% formaldehyde fixation. The EdU staining was performed following the manufacturer’s instructions (BCK-EDU488, Sigma, Saint Louis, MO, USA). The nuclei were stained with 2.5 mg/mL DAPI (Sigma) for 10 min. Following PBS washes, the cover-slip were mounted on microscope slides with Mowiol. The observations were performed by confocal microscopy by keeping the photomultiplier at a constant gain (Leica SP5, Leica Microsystems, Wetzlar, Germany). ImageJ software was used to quantify the green signal intensity in each nucleus. Each cell with an integrated density value above 1 was scored as positive. At least three independent experiments were performed, and data are presented as mean ± 1 SD.

### 2.5. RNA Extraction and RT-qPCR

Three and six days post-irradiation, total RNA was isolated from cells with ReliaPrep™ RNA Tissue Miniprep System (Promega, Z6111, Madison, WI, USA) according to the manufacturer’s instructions. RNA concentration was quantified using the Nanophotometer N60 (Implen, Munich, Germany). cDNA was synthetized with GoScript™ Reverse Transcription Mix (Promega, A2790) according to the manufacturer’s instructions using random primers. 2 μg of RNA was used per reaction. The primers used for the qPCR are described in Table S1. Linear relationships between Ct values and cDNA concentration expressed in log2 were checked for all primer pairs. All real-time PCR reactions were performed in duplicates with GoTaq™ qPCR Master Mix (Promega, A6001) on a ViiA 7 Real-Time PCR System (Applied Biosystems, Waltham, MA, USA) using a standard run. The gene expression level of each messenger RNA (mRNA) was calculated, further normalized to glyceraldehyde 3 phosphate dehydrogenase (GAPDH) mRNA, and related to the control condition. Specific amplification was confirmed by melting curve analysis. At least three independent experiments were performed, and data are presented as mean ± 1 SD.

### 2.6. Measurement of IL6 Secretion

Secreted IL6 was assessed in cell-free supernatants collected 6 days after irradiation using ELISA kit, according to the manufacturer’s recommendations (Quantikine, R&D Sytems D6050, Minneapolis, MN, USA). For all samples, measured concentrations were normalized by the number of viable cells. At least three independent experiments were performed, and data are presented as mean ± 1 SD.

### 2.7. Flow Cytometry

The apoptotic fraction was assessed with a FITC Annexin V detection kit (BD Pharmignen, Franklin Lakes, NJ, USA, No. 556547) 72 h after irradiation. The samples (composed of both cells present in the supernatant and adherent to the bottom of the wells) were handled following the manufacturer’s instructions. Data acquisitions were performed using FACS Verse (BD Biosciences, Franklin Lakes, NJ, USA) and quantifications using FlowJo software. Based on the intensity of Annexin V and propidium iodide fluorescence, the proportion of dead cells can be determined. Annexin V positive cells were considered as dead cells. The PI fluorescence intensity of Annexin V positive cells differentiated early apoptosis (PI negative) to late apoptosis or post-apoptotic necrosis (PI positive). At least three independent experiments were performed, and data are presented as mean ± 1 SD.

### 2.8. Colony Formation Assay and Survival Fraction

After irradiation, cells were detached using trypsin and seeded in 12-well plates at desired concentrations in medium (with or without 0.5 µM Olaparib) supplemented with 10% FBS. More precision on the procedure can be found in [38,39]. After 24 h, the medium was refreshed with FBS-supplemented medium containing or not ABT-263 at 1 µM and cells were left to proliferate (8 days for KP4 cells and 12 days for A549 and HCT-116 cells). The cells were then stained with crystal violet in 2% ethanol and the number of visible colonies (containing more than 50 cells) was counted. The plating efficiency (PE) was calculated for each irradiation dose and drug condition as the ratio of the number of colonies to the number of cells seeded. The survival fraction at a dose D is then obtained with comparison to un-irradiated cells:

(1) $${\mathrm{SF}}_{D}=\frac{PE_{D}}{PE_{0}}$$

With *PE_D_* and *PE*_0_ the plating efficiency calculated at a dose *D* and 0 Gy (no irradiation), respectively. Survival fraction can be calculated from un-irradiated cells that have not been in the presence of neither Olaparib nor ABT-263 (CTL cells) or from un-irradiated cells exposed to Olaparib, ABT-263 or both. At least three independent experiments were performed, and data are presented as mean ± 1 SD.

The coefficient of drug interaction (CDI) highlighting the potentiation obtained when combining two drugs can be calculated from the survival fractions:

(2) $$\mathrm{CDI}=\frac{SF_{Ola+ABT}}{SF_{Ola}\times SF_{ABT}}\times SF_{CTL}$$

where SF is the survival fraction associated with a chosen dose in the case of control, i.e., no drugs, (*SF_CTL_*), Olaparib (*SF_Ola_*), ABT-263 (*SF_ABT_*) and the combination Olaparib plus ABT-263 (*SF_Ola_*_+*ABT*_). These SF are calculated from the corresponding un-irradiated cells (CTL, ABT-263, Olaparib or both). A CDI below 0.9 considers the combination as synergic, between 0.9 and 1.1 as additive and above 1.1 as antagonist.

The amplification factor (AF) at a dose *D* is calculated as:

(3) $${\mathrm{AF}}_{D}=\frac{SF_{D,CTL}-SF_{D,\;drug}}{SF_{D,CTL}}\times 100\;\left(\%\right)$$

where *SF_D_*_,*CTL*_ is the survival fraction at dose *D* without either Olaparib nor ABT-263 and *SF_D_*_,*drug*_ corresponds to the survival fraction at dose *D* of cells exposed to either Olaparib, ABT-263 or both. The amplification factor highlights the increase in cell death when using drugs compared to irradiation alone.

### 2.9. Statistical Analysis

Statistical analyses were performed with GraphPad Prism. The unpaired two-tail Student *t*-test was applied considering that the data were normally distributed. If the variance between groups were not similar, Welch’s correction was applied. The groups compared are CTL vs. 1 µM ABT-263, CTL vs. 0.5 µM Olaparib and 0.5 µM Olaparib vs. 0.5 µM Olaparib plus 1 µM ABT-263. All experiments were repeated at least three times (*n* = 3).

## 3. Results

### 3.1. ABT-263 Hampers the Senescence-Promoting Effect of Olaparib after X-ray and Proton Radiation in A549 Cells

**Figure 1:**
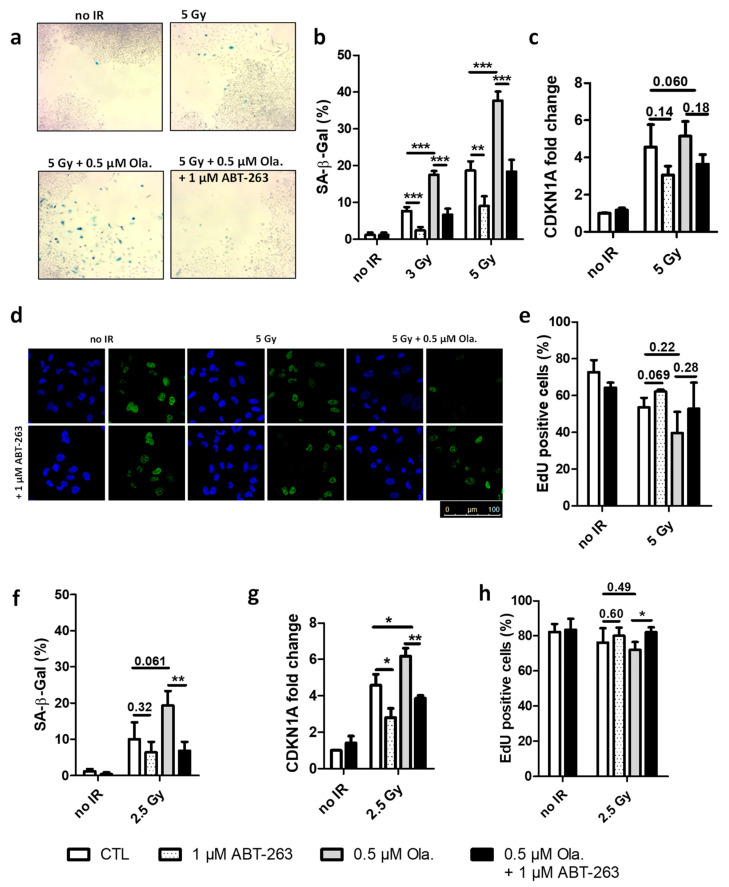
ABT-263 hampers the senescence-promoting effect of Olaparib after X-ray and proton radiation in A549 cells. A549 cells were irradiated with 3 and 5 Gy X-rays or 2.5 Gy protons with or without 0.5 µM of Olaparib; The medium was replaced 22 h after irradiation, and 1 µM of ABT-263 was added. (**a**) Bright field microscopic images were obtained 12 days after X-ray irradiation. Blue cells are positive for senescence associated beta-galactosidase activity. (**b**,**f**) Quantification of SA-β-Gal positive cells 6 days after irradiation ((**b**): X-rays; (**f**): protons). (**c**,**g**) CDKNA1 mRNA level 3 days after treatment ((**c**): X-rays; (**g**): protons). (**d**) Representative confocal images of A549 cells 6 days post-irradiation after an 8 h pulse of 10 µM EdU: nucleus (blue), EdU (green). (**e**,**h**) Quantification of EdU incorporation after irradiation ((**e**): X-rays; (**h**): protons). At least three independent experiments were performed, and data are presented as mean ± 1 SD. Unpaired *t*-tests were performed (*: *p* < 0.05; **: *p* < 0.01; ***: *p* < 0.001) for CTL vs. ABT, CTL vs. Ola and Ola vs. Ola + ABT.

A549 cells were irradiated with X-rays at 3 and 5 Gy or with 1.3 MeV protons at 2.5 Gy. 2 h before irradiation, 0.5 µM Olaparib was added and left for 24 h. ABT-263 (1 µM) was then added and 3 to 12 days post-irradiation, cells were analyzed for senescence markers (Figure 1). These concentrations of Olaparib and ABT-263 led to limited (although significant) toxicity in un-irradiated cells with survival close to that of the control cells (Table S2).

The induction of senescence was investigated in A549 cells exposed to irradiation with or without these different drugs. For that purpose, SA-β-Gal activity was evaluated, EdU incorporation (indicating DNA synthesis) was assessed and CDKN1A mRNA level (related to cell cycle arrest) was determined. Figure 1a presents pictures of SA-β-Gal staining in cells obtained at 12 days after 5 Gy X-rays (end time for clonogenic survival assays). The presence of senescent cells after irradiation was observed, especially if Olaparib was used. These senescent cells in between colonies were no longer observed in the presence of ABT-263. Quantifications of SA-β-Gal activity in A549 cells 6 days after irradiation are presented in Figure 1b, showing that X-ray irradiation induced an increase in the proportion of senescent cells in a dose-dependent manner and that Olaparib significantly increased it further. Moreover, the addition of ABT-263 reduced the proportion of senescent cells down to the level of the one observed for irradiation alone. At 3 days, the mRNA level of CDKN1A was increased after X-ray irradiation and was reduced in the presence of ABT-263 (Figure 1c, data at day 6 post-irradiation presented in Supplemental Figure S1a). Figure 1d displays confocal images of A549 cells exposed to an 8 h pulse of 10 µM EdU 6 days post-irradiation. A decreased incorporation in irradiated cells and a recovery in the presence of ABT-263 were observed. The quantification of EdU incorporation (Figure 1e) showed a reduced cell proliferation after irradiation. As for SA-β-Gal activity, Olaparib treatment accentuated this effect. In this case also, ABT-263 partly relieved the induction of cell cycle arrest. As it was reported by others in senescent cells [42], a nuclear enlargement was also observed after irradiation (Supplemental Figure S1c). It was reduced in the presence of ABT-263.

Although to a lesser extent, similar results were obtained after 2.5 Gy proton irradiation with increased SA-β-Gal activity (Figure 1f). A higher level of CDKN1A mRNA (Figure 1g, data for day 6 in Supplemental Figure S1b), reduced EdU incorporation (Figure 1h) and nuclear enlargement (Supplemental Figure S1d) were observed. The increase in CDKN1A mRNA level was still significant for the proton at 6 days. As for X-rays, the combination of Olaparib with proton irradiation strengthened the senescent phenotype and ABT-263 was able to counteract the effect of Olaparib.

Together, these results showed that Olaparib promoted the senescence induced by X-ray and proton radiation in A549 cells and that ABT-263 was able to revert it.

### 3.2. The Effect of ABT-263 Is Lower for Cell Lines with Limited Radio-Induced Senescence

**Figure 2:**
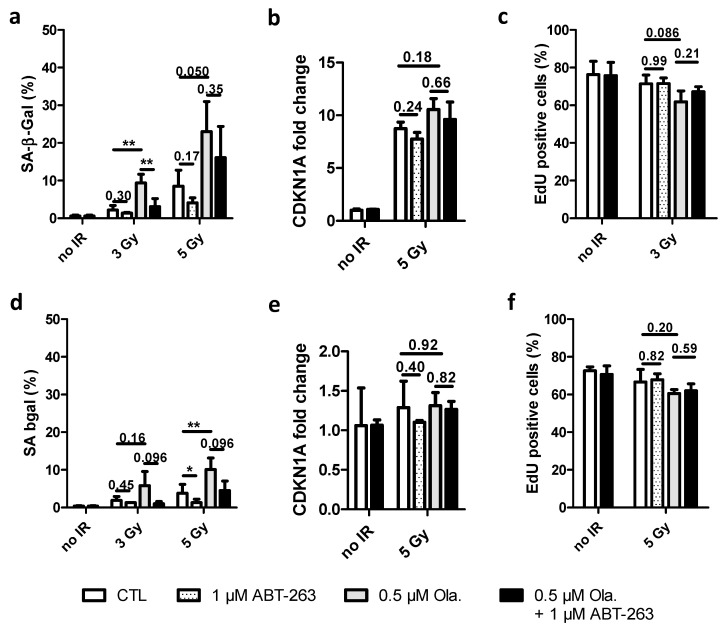
The effect of ABT-263 is reduced for cell lines with limited radio-induced senescence. HCT-116 and KP4 cells were irradiated with 3 and 5 Gy X-rays with or without 0.5 µM of Olaparib. The medium was replaced 22 h after irradiation, and 1 µM of ABT-263 was added. (**a**,**d**) Quantification of SA-β-Gal positive cells 6 days after irradiation ((**a**): HCT-116; (**d**): KP4). (**b**,**e**) CDKNA1 mRNA level 3 days after treatment ((**b**): HCT-116; (**e**): KP4). (**c**,**f**) Quantification of EdU incorporation after a 10 µM 8 h pulse ((**c**): HCT-116; (**f**): KP4). At least three independent experiments were performed, and data are presented as mean ± 1 SD. Unpaired *t*-tests were performed (*: *p* < 0.05; **: *p* < 0.01) for CTL vs. ABT, CTL vs. Ola and Ola vs. Ola + ABT.

HCT-116 and KP4 cells were irradiated with 3 and 5 Gy X-rays. Two hours before irradiation, 0.5 µM Olaparib was added for 24 h. ABT-263 (1 µM) was then added and 6 days post-irradiation, cells were analyzed for senescence markers (Figure 2). These concentrations of Olaparib and ABT-263 led to limited toxicity (although significant for HCT-116 cells) with cell survival without irradiation close to the one of control cells (Table S2).

For HCT-116 cells, the proportion of SA-β-Gal positive cells, even if lower than for A549 cells, also increased with Olaparib (~25% at 5 Gy vs. ~40% for A549 cells) (Figure 2a). The CDKN1A mRNA level markedly increased 3 days after 5 Gy irradiation with slight effects on Olaparib and ABT-263 (Figure 2b). At 6 days, the CDKN1A mRNA level was diminished (Supplemental Figure S2a). The EdU incorporation assay (Figure 2c) followed the same trend. The addition of ABT-263 reduced the proportion of senescent cells in HCT-116 cells, but to a lesser extent than what was observed for A549 cells. The enlargement of the nuclear area varied accordingly (Supplemental Figure S2c). For KP4 cells, SA-β-Gal activity moderately increased with irradiation and Olaparib (Figure 2d). In agreement with the low proportion of SA-β-Gal positive cells (10% at 5 Gy), CDKN1A mRNA levels were very slightly increased after irradiation (Figure 2e and Supplemental Figure S2b at 6 days). As for HCT-116 cells, EdU (Figure 2f) labeling in irradiated cells was close to the one of un-irradiated cells and the exposure to Olaparib and/or ABT-263 did not lead to notable changes, as it was noticed for nuclear enlargement (Supplemental Figure S2d).

These results showed that although Olaparib promoted the senescence induced by X-ray irradiation in HCT-116 and KP4 cell lines, the induction of senescence is cell line-dependent with a lower effect in HCT-116 and KP4 cells compared to the pro-senescence phenotype observed in A549 cells.

### 3.3. ABT-263 Induces Cell Death in Cell Lines Displaying Radiation-Induced Senescence

**Figure 3:**
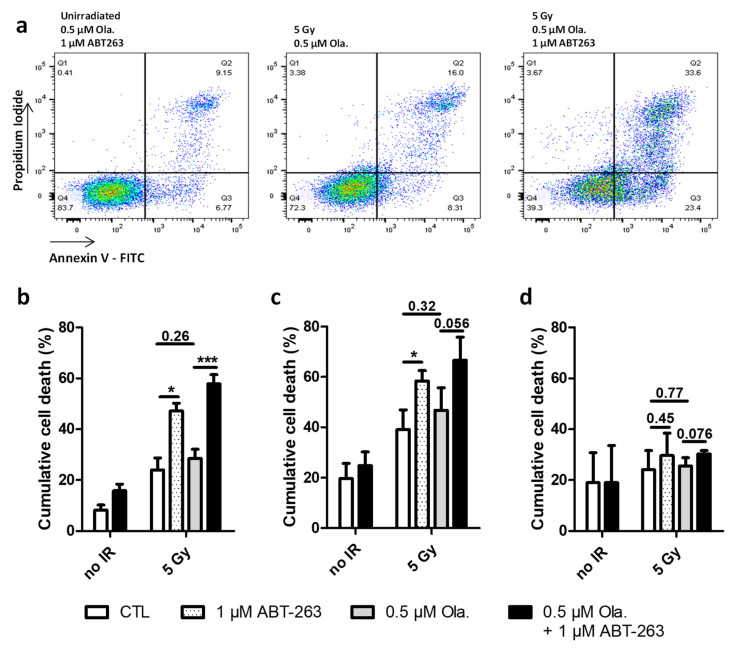
ABT-263 can induce cell death in cell lines displaying radiation-induced senescence. A549, HCT-116 and KP4 cells were irradiated with 5 Gy X-rays with or without 0.5 µM of Olaparib. The medium was replaced 22 h after irradiation, and 1 µM of ABT-263 was added. (**a**) Representative flow cytometry results for Annexin V assessment in A549 cells 72 h after irradiation. (**b**–**d**) Cumulative cell death 72 h after X-rays ((**b**): A549 cells; (**c**): HCT-116 cells, (**d**): KP4 cells). At least three independent experiments were performed, and data are presented as mean ± 1 SD. Unpaired *t*-tests were performed (*: *p* < 0.05; ***: *p* < 0.001) for CTL vs. ABT, CTL vs. Ola and Ola vs. Ola + ABT.

Cumulative cell death was measured by flow cytometry 72 h after 5 Gy X-ray irradiation for A549, HCT-116 and KP4 cells (Figure 3). Representative distributions for A549 cells are presented in Figure 3a. The proportion of Annexin V positive cells was quantified for each cell line. For A549 cells (Figure 3b), irradiation alone or irradiation combined with Olaparib led to a slight increase in cumulative cell death. The Annexin V positive fraction significantly increased in the presence of ABT-263. The highest percentage, reaching approximately 60% of dead cells, was observed when Olaparib and ABT-263 were combined after irradiation. The level of cumulative cell death in HCT-116 cells (Figure 3c) after irradiation was higher compared to A549 cells and the effect of ABT-263 was also highlighted but to a lower extent than for A549 cells. For KP4 cells, on the other hand, the fraction of dead cells in irradiated cells only slightly increased compared to un-irradiated cells and no effect of Olaparib, ABT-263 or their combination was noticeable (Figure 3d). For A549 cells, the distribution between early and late apoptosis was about 50–50%, while for HCT and KP4 cell lines, dead cells were mostly in late apoptosis or necrosis.

PARP cleavage was analyzed by Western blot 6 days post-irradiation in A549 cells exposed to 5 Gy X-rays (Supplemental Figure S3a,b). The ratio of the cleaved over full length forms of PARP was higher in irradiated cells in the presence of ABT-263, especially when combined with Olaparib. A slight increase in Bcl-XL mRNA levels 3 and 6 days after X-ray or proton irradiation was observed in the presence of Olaparib (Supplemental Figure S3c–f). In both cases, the addition of ABT-263 was able to reduce, to a small extent, this increase.

These results showed that ABT-263 was able to promote cell death after irradiation in cell lines displaying radiation-induced senescence.

### 3.4. Non-Toxic Combination of ABT-263 and Olaparib Can Synergize after X-ray and Proton Irradiation to Increase Clonogenic Cell Death

**Figure 4:**
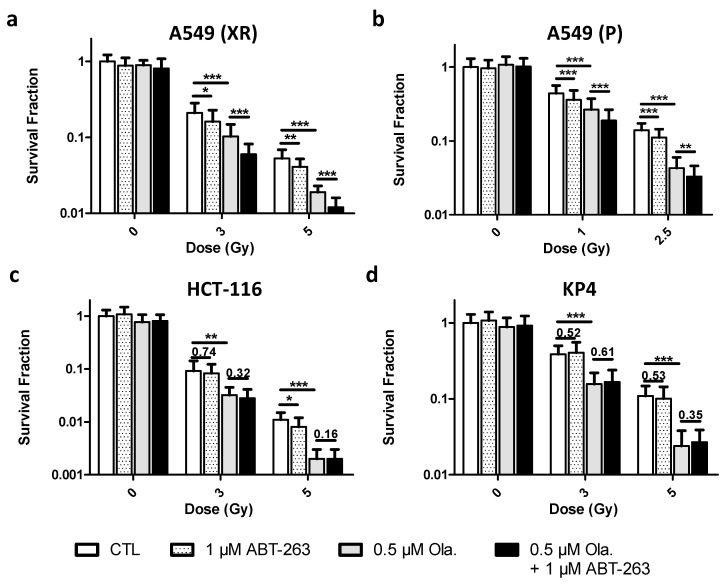
Non-toxic combination of ABT-263 and Olaparib can synergize after X-ray and proton irradiation to increase clonogenic cell death. A549, HCT-116 and KP4 cells were irradiated with 3 and 5 Gy X-rays or 1 and 2.5 Gy protons with or without 0.5 µM of Olaparib. The medium was replaced 22 h after irradiation, and 1 µM of ABT-263 was added. Colonies were stained and counted 8 days (KP4 cells) or 12 days (A549, HCT-116 cells) post-irradiation. (**a**) A549 cells after X-rays. (**b**) A549 cells after protons. (**c**) HCT-116 cells after X-rays. (**d**) KP4 cells after X-rays. At least three independent experiments were performed, and data are presented as mean ± 1 SD. Unpaired *t*-tests were performed (*: *p* < 0.05; **: *p* < 0.01; ***: *p* < 0.001) for CTL vs. ABT, CTL vs. Ola and Ola vs. Ola + ABT.

Clonogenic fractions were assessed after 3 and 5 Gy X-ray irradiation in A549, HCT-116 and KP4 cell lines as well as after 1 and 2.5 Gy proton irradiation for A549 cells (Figure 4). For all cell lines, the combination of Olaparib and ABT-263 led to limited toxicity in un-irradiated cells as shown in Figure 4 (values are presented in Table S2). HCT-116 cells were more radiosensitive than A549 and KP4 cells.

For A549 cells, after X-ray irradiation, a decrease in survival fraction upon the addition of Olaparib was observed for each irradiation dose (Figure 4a). While the addition of ABT-263 significantly decreased survival, the combination with Olaparib reduced survival to a much higher extent compared to Olaparib and ABT-263 alone. The same observation was made after proton irradiation (Figure 4b), with a pronounced radiosensitization when Olaparib and ABT-263 were combined. For HCT-116 cells (Figure 4c), a decrease in the survival fraction with the irradiation dose and with the addition of Olaparib was observed. The addition of ABT-263 did not further increase the clonogenic death. KP4 cells were radiosensitized by Olaparib but the combination with ABT-263 did not lead to a higher clonogenic cell death (Figure 4d).

The interaction of Olaparib and ABT-263 was characterized using the coefficient of drug interaction (CDI) and the amplification factor (AF) (Table 1). For A549 cells, a synergistic effect was obtained in most cases, with an increased AF for the combination of the drugs compared to Olaparib alone. For HCT-116 cells, the CDI calculated corresponded to an additive effect of the drugs with a slightly increased AF for the combination compared to Olaparib alone. For KP4 cells, CDI and AF did not highlight a potentiation of the drugs when combined. Moreover, an antagonist effect was calculated at 5 Gy.

These results showed that Olaparib sensitized A549, HCT-116 and KP4 cells to radiation. ABT-263 alone slightly increased cell death after X-rays or protons compared to Olaparib. ABT-263 and Olaparib can synergize in A549 cells to reduce clonogenic cell death after X-ray and proton irradiation. For HCT-116 cells, the combination was slightly less efficient than for A549 cells. For KP4 cells, the combination was not effective with a CDI above 1.1 at 5 Gy and a smaller amplification factor compared to Olaparib alone.

### 3.5. ABT-263 Reduces the SASP Induced by Olaparib after X-ray and Proton Irradiation in A549 Cells

**Figure 5:**
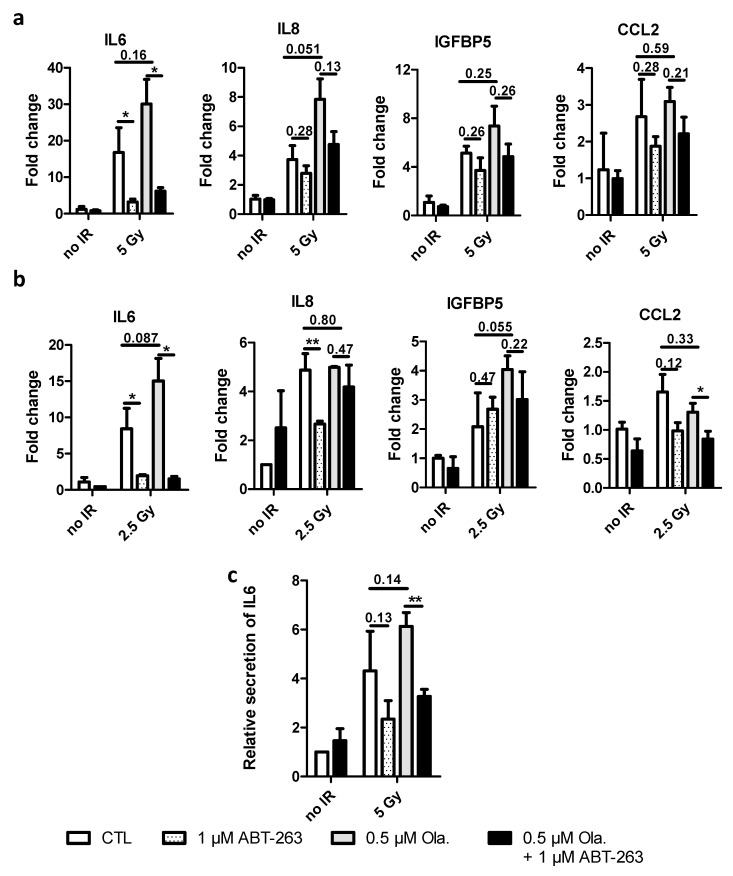
ABT-263 reduces the SASP promoted by Olaparib and radiations in A549 cells. A549 cells were irradiated with 5 Gy X-rays or 2.5 Gy protons with or without 0.5 µM of Olaparib. The medium was replaced 22 h after irradiation, and 1 µM of ABT-263 was added. (**a**,**b**) 3 days post-irradiation, mRNA level of genes implicated in SASP were determined. Fold changes, calculated as 2^−ΔΔct^, are presented after X-rays (**a**) and protons (**b**) radiation. (**c**) Relative secretion of IL6 six days after X-rays. At least three independent experiments were performed, and data are presented as mean ± 1 SD. Unpaired *t*-tests were performed (*: *p* < 0.05; **: *p* < 0.01) for CTL vs. ABT, CTL vs. Ola and Ola vs. Ola + ABT.

Three days after irradiation, mRNA levels for IL6, IL8 (CXCL8), IGFB5 and CCL2 (MCP1) were determined in A549 cells exposed to X-rays (Figure 5a) and protons (Figure 5b). These genes have been reported as genes associated with the SASP and are upregulated upon stress-induced senescence [33,42]. Results at 6 days are presented in Supplemental Figure S4. The levels of mRNAs in these genes increased with radiation (photons and protons). The addition of Olaparib seemed to further increase mRNA levels but not significantly. ABT-263 was able to lower this effect, especially for IL6. As for the level of SA-β-Gal activity, the induction of SASP genes after 2.5 Gy is smaller than 5 Gy X-rays.

The results obtained for the mRNA levels for IL6 were confirmed at protein level (Figure 5c). Six days post-irradiation, the level of IL6 in the culture medium was higher for X-ray irradiated cells compared to un-irradiated cells. The presence of Olaparib enhanced this effect. The addition of ABT-263 22 h after irradiation was able to reduce it.

The mRNA levels of these genes were also assessed in HCT-116 and KP4 cells after 5 Gy X-rays (Supplemental Figure S5). The effect of irradiation was limited for most genes and, as expected, because of the lower induction of senescence and the lower effect of ABT-263: the responses to Olaparib or ABT-263 were rather small and not observed for all genes.

## 4. Discussion

In this work, we proposed taking advantage of the senolytic drug, ABT-263, to enhance the effects of the combination of radiation (X-rays or protons) and a PARPi, Olaparib.

Even though the use of Olaparib is currently recommended for cancer patients with germline BRCA mutations, PARPi have been demonstrated to be efficient as sensitizing agents in cell lines with BRCA1/2 wild-type tumors. In this work, HR proficient cell lines have been studied, namely A549, HCT-116 and KP4 cells. These three cell lines are TP53 wild-type and KRAS-mutated cancer cells. A549 and HCT-116 cells are CDKN2A deficient. Previous works from our team and others showed that both A549 and HCT-116 cells can be radiosensitized by Olaparib and undergo senescence after irradiation. On the other hand, KP4 cells are not known to display a stress-induced senescent response. The increase in senescence following irradiation and treatment with PARPi has already been reported [22,24,43]. Recently, Fleury et al. also demonstrated that ovarian and breast cancer cells treated with Olaparib acquired a senescent-like phenotype [21]. The mechanisms of action regarding the induction of senescence following the inhibition of PARP are not well known. However, it was demonstrated that the induction of p21 is necessary for senescence to occur following treatment with PARPi [21]. Several high-grade serous epithelial ovarian cancer cells (HGSOC) were treated with PARPi, namely Olaparib, Talazoparib and Niraparib. These molecules induced a senescence-like phenotype in these cells, meaning the findings are likely not limited to Olaparib [21]. Senescence has often been seen as a double-edged sword in cancer [29,33]. For this reason, we proposed to eliminate these senescent cells using senolytic drugs such as ABT-263, an inhibitor of Bcl2, Bcl-x_L_ and Bcl-w in order to enhance treatment efficacy.

We showed that X-ray irradiation induced a cell line-dependent senescence. In addition, Olaparib further enhanced the X-ray-induced senescent phenotype. Moreover, our results demonstrated that the use of ABT-263 was able to counteract the senescence-promoting effect of Olaparib after irradiation. The literature on proton irradiation induced senescence is limited. However, we confirmed here our previous results and we showed that proton irradiation, at a LET of 25 keV/µm, induced senescence [19]. The induction of senescence by protons was also recently observed by Schniewind et al. [20]. These authors exposed glioblastoma, prostate and head-and-neck cancer cells to 3.7 keV/µm protons and photons. They observed that 4 Gy irradiation led to an increased SA-β-Gal activity for both types of radiations. For prostate cancer and glioblastoma cells, the induction was less pronounced after proton irradiation compared to photon irradiation. It would be interesting to study whether the radiation quality (using particles of various LETs) could influence the induction of senescence. In this work, as for photons, the combination with Olaparib enhanced the proton-induced senescent phenotype and the addition of ABT-263 prevented this effect.

p21 (CDKN1A) is an inhibitor of cyclin-dependent-kinases and its induction by p53 leads to cell cycle arrest in G1 due to the inhibition of CDK4 and CDK6, while it also prevents G2/M transition initiated by CDK1-cyclinB complex. It is already known that the induction of senescence by PARPi and radiation is mediated by p21 (CDKN1A) [21]. Our results showed that the mRNA expression of CDKN1A was increased following combined treatments (PARPi and radiation), while the use of ABT-263 decreased the mRNA expression of CDKN1A. Our results also indicate that the effect of this senolytic drug depends on the ability of radiation and PARPi to induce senescence. Indeed, the effect of ABT-263 was lower in KP4 cells, as the percentage of SA-β-Gal positive cells only reached 10% following radiation and PARP inhibition. This is also confirmed by the lack of cells in between colonies (data not shown) that were observed for A549 cells (Figure 1a). The lower induction of senescence could be explained by the fact that the Chk2-p53-p21 pathway may not have been activated since we did not observe a significant increase in mRNA expression of CDKN1A following radiation and PARP inhibition in KP4 cells. However, the induction of p21 is a dynamic process that can determine cell fate [44]. In this later work, the authors followed, at a single cell level, the induction of p21 in A549 and HCT-116 cells before, during and days after (up to 96 h) drug treatment. They highlighted three different patterns: (i) cells exhibiting early and transient acute p21 response (level back to control within 36 h) were found to proliferate, (ii) cells exhibiting an early acute high level of p21 maintained over time became senescent, (iii) cells exhibiting a delayed but increasing (from 24 h) induction of p21 over time became senescent. The first two patterns were found in G1 cells while the last was associated with S/G2 cells. Hsu and colleagues showed that ATM signaling is required for high levels of drug-induced p21 expression in G1. Regarding the unexpectedly lower levels of p21 expression in S/G2, they evidenced a p21 expression repression mediated by Chk1 signaling and proteasomal degradation [44]. The low level of CDKN1A mRNA in KP4 cells 72 h after treatment could indicate an early and transient induction of p21 that is associated with proliferation and not to senescence as proposed in Hsu’s work.

The induction of senescence after radiation (with or without Olaparib) was also observed in TP53-mutated MDA-MB-231 cells (Supplemental Figure S6a). As for A549 cells, Olaparib promoted SA-β-Gal activity after irradiation. Although ABT-263 led to a strong reduction of SA-β-Gal activity, no effect of Olaparib or of ABT-263 was detected on the induction of CDKN1A mRNA level at 3 days (Supplemental Figure S6b). At day 6 (Supplemental Figure S6c), the mRNA level of CDKN1A was higher for cells irradiated in the presence of Olaparib, but again, the effect of ABT-263 was negligible. In TP53 mutant cells, Chk2 is responsible for p21 induction and senescence [45]. Despite a possible correlation between the induction of cell death (Annexin V positive cells) and the reduction of SA-β-Gal positive cells proportion with the addition of ABT-263 when considering A549, HCT-116 and KP4 cells, no remarkable difference in cell death quantification was observed in MDA-MB-231 cells with the addition of ABT-263 (Supplemental Figure S6d). Still, the implication of p21 in apoptosis has been evidenced by others and notably for A549 and HCT-116 cells [46,47]. These authors showed that downregulation of p21 using p21 antisense oligodeoxynucleotide led to increased apoptosis. Here, we observed, at 72 h, a strong correlation between the increase in Annexin V positive cells and the reduction of CDKN1A mRNA level when ABT-263 was added (Supplemental Figure S7). The low decrease in CDKN1A mRNA level of MDA-MB-231 cells with the addition of ABT-263 could thus explain the lack of Annexin V positive cells in comparison to A549 cells.

We demonstrated that further sensitization by ABT-263 is correlated with the ability of the treatment to induce senescence. As presented above, the use of ABT-263 alone did not induce immediate nor clonogenic cell death, indicating that this drug specifically targets senescent cells. While A549, HCT-116 and KP4 cells were sensitized by Olaparib after X-ray or proton irradiation, the combination with ABT-263 led to a synergic increased cell death only for A549 cells. For KP4 cells, with very limited radio-induced senescence, the combination of ABT-263 with Olaparib did not reduce survival fraction. For MDA-MB-231 cells, the radiosensitizing effect of Olaparib was limited while ABT-263 significantly affected cell survival (Supplemental Figure S6e). This could be due to the lack of p53 in these cells. Nonetheless, as for A549 cells, the combination of ABT-263 with Olaparib led to a synergistic effect upon 5 Gy X-ray irradiation (CDI = 0.87). This synergistic effect is in agreement with SA-β-Gal activity results. Furthermore, a correlation was observed between the reduction in SA-β-Gal activity with the addition of ABT-263 and the calculated CDI as shown in Supplemental Figure S8.

The release of SASP factors is able to modulate several aspects, including proliferation, metastasis, treatment resistance and immunosuppression. SASP factors can induce epithelial–mesenchymal transition (EMT) as reported by Coppé et al. [48]. It was also evidenced that IL6 and IL8 can promote proliferation through the activation of STAT3 [49,50]. The activation of the latest STAT3 regulates c-myc, c-Fos, cyclin D1 and mammalian target of rapamycin complex 1 (mTORC1) expression. Moreover, these pro-tumorigenic SASP factors have also been implicated in the regulation of invasive properties of cancer cells. Cancer cells incubated with a conditioned medium of senescent cells demonstrated a higher capacity to invade the basement membrane [48]. IL6 and IL8 blocking antibodies reduced the invasion capability of the cells exposed to these conditioned media. Our results demonstrated that irradiation (X-rays and protons), and notably in the presence of Olaparib, led to increased mRNA levels of SASP genes in A549 cells (Figure 5a,b, respectively) and MDA-MB-231 cells (Supplemental Figure S6f). This was especially the case for IL6 and IL8 mRNAs. The incubation in the presence of ABT-263 decreased IL6 and IL8 mRNA expression. The decrease in IL6 mRNA expression was confirmed at the protein level for A549 cells. Overall, these results demonstrate that ABT-263 can successfully decrease the expression of SASP factors and could ultimately prevent its pro-tumorigenic role.

## 5. Conclusions

Our results constitute a first step to further preclinical investigation in order to combine PARP inhibitors to proton or photon irradiation and inhibitors of the Bcl-2 or Bcl-xL family. Clinical limitations of this strategy are risks for ABT-263 to induce side effects such as thrombocytopenia and neutropenia [51]. Nonetheless, a study has mentioned the safety of ABT199 (Venetoclax), a Bcl-2 inhibitor [52]. Furthermore, the proteolysis-targeting chimera (PROTAC) technology was recently used to decrease the platelet toxicity of ABT-263 [53].

We worked with BRCA1/2 will type cells and demonstrated that both TP53 wild-type and mutant cell lines could be targeted by this strategy as long as senescence induction is observed. More work and more cell lines are needed to further characterize the effect of Olaparib and ABT-263 on radio-induced senescence.

The concentration of Olaparib (0.5 µM) and ABT-263 (1 µM) chosen aimed at limited toxicities in un-irradiated cells even though higher doses should lead to stronger effects. Furthermore, studying the establishment of senescence, in the presence or not of Olaparib, could point out a better schedule for the combination. For that purpose, single cell analysis using live cell imaging could be of particular interest. In our case, the cells were incubated 24 h with Olaparib before it was replaced by ABT-263. A longer incubation time and even a co-incubation could be of interest.

This study could be extended to other senolytics. Indeed, in the context of senescence, it was demonstrated that several anti-apoptotic pathways are upregulated. A senolytic panel composed of dasatinib, querceting, fisetin and piperlongumine (PPL) has already been used in combination with Olaparib in HGSOC cells. The authors demonstrated that synergy occurred between Olaparib and the different senolytics that were tested [21]. Interestingly, ABT-263, A-1155463 and PPL were also tested in combination with X-rays or Olaparib in LNCaP and PC-3 cells. Combining ABT-263 or A-1155463 to Olaparib or X-rays increased cell death in LNCaP and PC-3 cells. However, it was not the case for PPL supporting the fact that the upregulation of anti-apoptotic genes following senescence depends on the inducer and on the cell type [23].

To conclude, the use of such senescence-targeting inhibitors could enhance the effects of combining PARP inhibitors to conventional or proton radiotherapy and prevent adverse effects associated with senescence induction.

## Figures and Tables

**Table 1:** Coefficient of drug interaction and amplification factor calculated with the survival fraction of A549, HCT-116 and KP4 cells after irradiation with 3 and 5 Gy X-rays (XR) or 1 and 2.5 Gy protons (P). The survival fractions were evaluated in comparison to un-irradiated cells.

		CDI			AF		
					1 µM ABT-263	0.5 µM Olaparib	Ola. + ABT-263
**A549 (XR)**	**3 Gy**	0.74	Syn.		13	46	65
	**5 Gy**	0.84	Syn.		13	61	71
**A549 (P)**	**1 Gy**	0.88	Syn.		15	43	58
	**2.5 Gy**	0.98	Add.		18	71	77
**HCT-116**	**3 Gy**	1.01	Add.		17	55	62
	**5 Gy**	1.08	Add.		31	72	79
**KP4**	**3 Gy**	1.04	Add.		2	54	53
	**5 Gy**	1.28	Anta.		14	76	73

## Data Availability

The data presented in this study are available upon request to the authors.

## Supplementary Materials

The following supporting information can be downloaded at: https://www.mdpi.com/article/10.3390/cancers14061460/s1, Table S1: Primers sequences. Figure S1: ABT-263 hampers the senescence-promoting effect of Olaparib after X-ray and proton irradiation in A549 cells. Figure S2: The effect of ABT-263 is reduced for cell lines with limited radio-induced senescence. Figure S3: ABT-263 induces cell death in cell lines displaying radiation-induced senescence. Table S2: Survival fraction of A549 cells exposed to 0.5 μM Olaparib and/or 1 μM ABT-263 without irradiation. Figure S4: ABT-263 reverts the SASP promoted by Olaparib after X-ray and proton irradiation in A549 cells. Figure S5: X-rays, Olaparib and ABT-263 effect on SASP genes in HCT-116 and KP4 cells. Figure S6: Induction of senescence in MDA-MB-231: effect of Olaparib and ABT-263. Figure S7: Correlation between increased Annexin V and CDKN1A fold change reduction. Figure S8: Correlation between CDI and reduction of SA-β-Gal by ABT-263. Reference [54] is cited in the supplementary materials.
