# Supplementary material for: Taking Advantage of the Senescence-Promoting Effect of Olaparib after X-ray and Proton Irradiation Using the Senolytic Drug, ABT-263
Source: Cancers (Basel). 2022 Mar 12;14(6):1460. doi: 10.3390/cancers14061460 (PMC8946554; doi:10.3390/cancers14061460)
Supplement: Supplementary file 1 [file cancers-14-01460-s001.zip › cancers-1607822-supplementary.pdf]

SUPPLEMENTAL INFORMATION

TAKING ADVANTAGE OF THE SENESCENCE PROMOTING EFFECT OF OLAPARIB AFTER X-RAY AND PROTON IRRADIATION USING THE SENOLYTIC DRUG ABT-263

Camille Huart [1], Maude Fransolet [1], Catherine Demazy [1], Benjamin Le Calvé [1], Stéphane Lucas [2], Carine Michiels [1] and Anne-Catherine Wéra [1,3*]

[1] Cellular Biology Research Unit (URBC) – Namur Research Institute For Life Sciences (NARILIS), UNamur, Belgium
[2] Laboratory of Analysis by Nuclear Reaction (LARN) – Namur Research Institute For Life Sciences (NARILIS), UNamur, Belgium
[3] Molecular Imaging, Radiation and Oncology (MIRO) Laboratory, UCLouvain, Belgium

MATERIAL AND METHODS

MDA-MB-231: Cell Culture, Olaparib and ABT-263

MDA-MB-231 cells were sub-cultured in RPMI medium supplemented with 10% FBS. For the experiments, the medium was supplemented with 0.1% penicillin/streptomycin (Sigma). Olaparib was used at 0.5 µM final concentration. ABT-263 was used at 1 µM final concentration. Cells were incubated in the presence of the Olaparib for total duration of 24 h, while ABT-263 was added 24 h after irradiation and was left up to the end of the assay.

Western blot analysis

Six days after irradiation, A549 cells were lysed as described in [1]. The western blot analyses were performed as described in [2]. Primary antibody against cleaved and full length PARP (9548, mouse, Bioke) was used at 1/1000 and β-actin (A5441, mouse, Sigma) at 1/10,000. Anti-mouse secondary antibody, IRDye 680RD and IRDye 800CW Goat anti-mouse IgG (Li-Cor Biosiences) were used at 1/10,000. The membranes were scanned with the Amersham Typhoon. β-actin was used as loading control. Three independent experiments were performed and data are presented as mean ± 1 SD.

RNA extraction and RT-qPCR

Table S1: Primer sequences

Primers	Forward sequence (5' - 3')	Reverse sequence (3' - 5')
CDKN1A	GTGGACCTGTCACTGTCTTG	GGCGTTTGGAGTGGTAGAAA
IL6	CCTGAACCTTCCAAAGATGGC	CACCAGGCAAGTCTCCTCATT
IL8	CTGGCCGTGGCTCTCTTG	GGGTGGAAAGGTTTGGAGTATG
IGFBP5	TGTGACCGCAAAGGATTCTACA	TCCCCGTCAACGTACTCCAT
CCL2	AAGTGTCCCAAAGAAGCTGT	TGGGTTGTGGAGTGAGTGTT
GAPDH	TGAAGGTCGGAGTCAACGG	GCAACAATATCCACTTTACCAGAGT

RESULTS

1. Combination of ABT-263 and Olaparib led to limited clonogenic cell death in un-irradiated cells.

Table S2: Survival fraction of A549 cells exposed to 0.5 µM Olaparib and/or 1 µM ABT-263 without irradiation. The survival fraction was evaluated related to unexposed cells. p values calculated with t-tests (unpaired with Welch's correction in case of significantly different variance).

	Survival fraction from CTL cells at 0 Gy		
	**1 µM ABT-263**	**0.5 µM Olaparib**	**Ola. + ABT-263**
**A549 (XR)**	0.88 ±0.24 (p=0.0675)	0.90 ±0.14 (p=0.0271*)	0.81 ±0.28 (p=0.0035**)
**A549 (p)**	0.96 ±0.28 (p=0.4844)	1.08 ±0.31 (p=0.1537)	1.02 ±0.30 (p=0.7629)
**HCt-116**	1.08 ±0.39 (p=0.4198)	0.77 ±0.29 (p=0.0192*)	0.80 ±0.25 (p=0.0153*)
**KP4**	1.08 ±0.32 (p=0.2467)	0.89 ±0.28 (p=0.0784)	0.92 ±0.32 (p=0.2516)

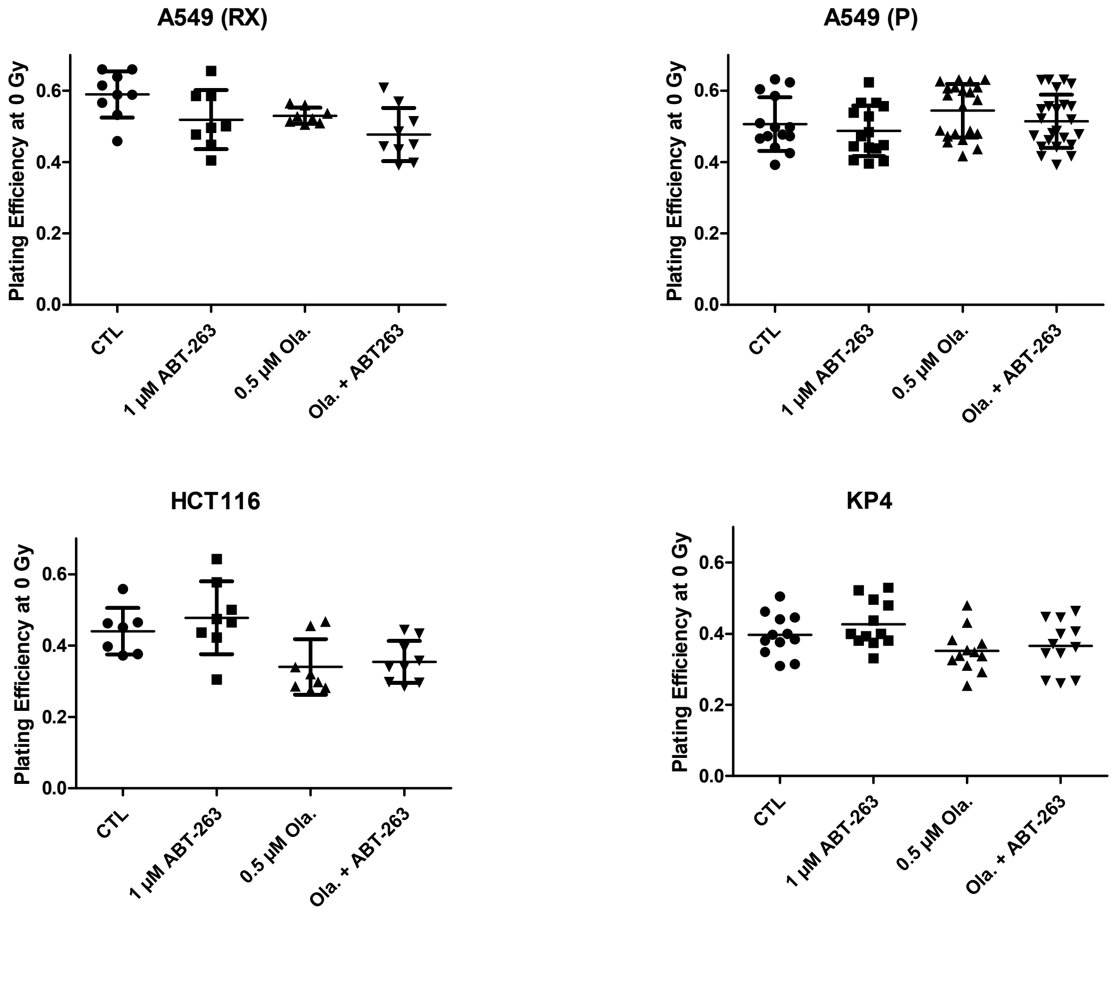

2. ABT-263 hampers the senescence inducing effect of Olaparib after X-ray and proton radiation in A549 cells

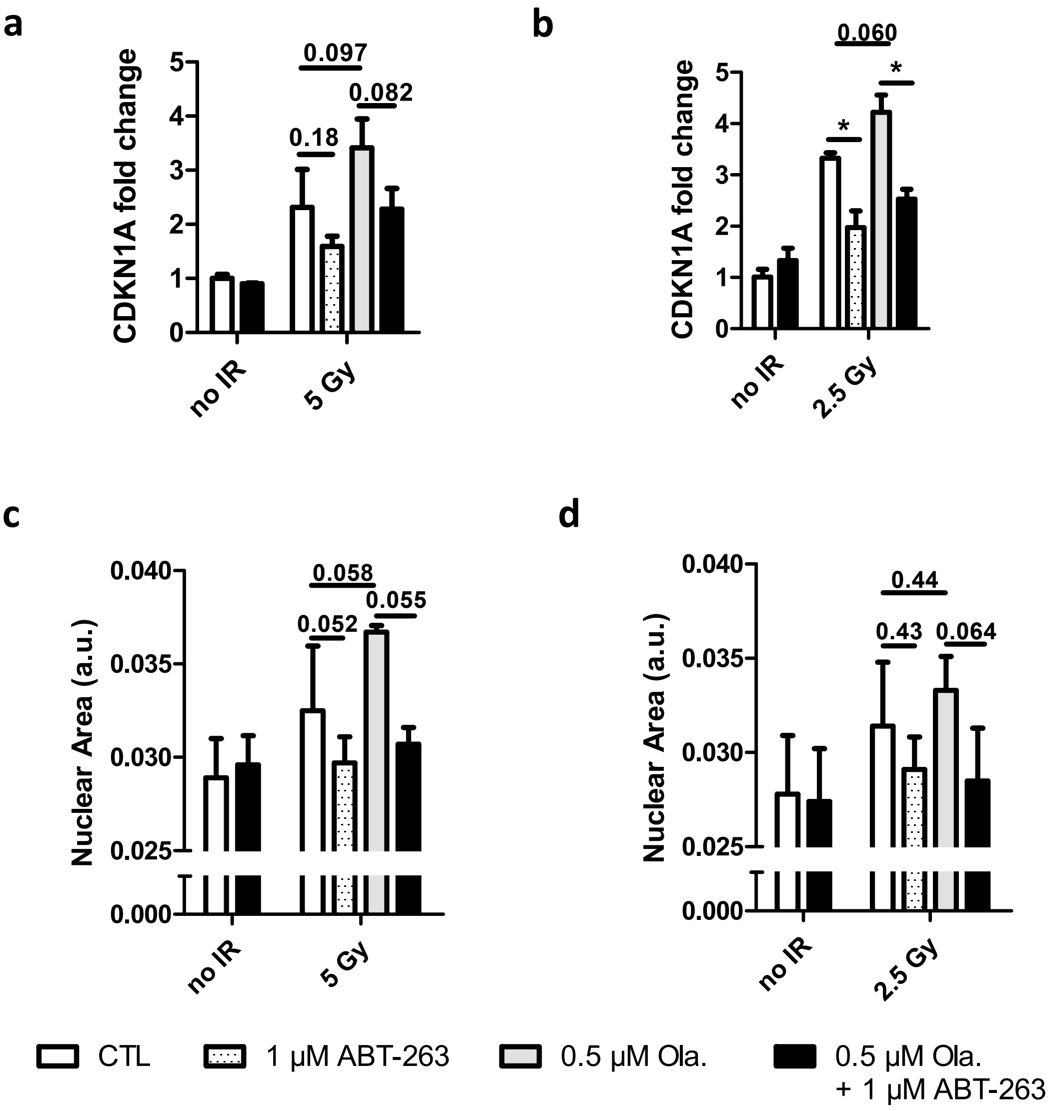

Figure S1: ABT-263 hampers the senescence promoting effect of Olaparib after X-ray and proton irradiation in A549 cells. A549 cells were irradiated with 5 Gy X-rays or 2.5 Gy protons with or without 0.5 µM of Olaparib. 22h after irradiation, the medium was replaced and 1 µM of ABT-263 was added. 6 days after irradiation, mRNA level of CDKN1A was evaluated (a: X-rays; b: protons). 6 days post-irradiation, cells were fixed and the nuclei stained with DAPI. The nucleus mean area of at least 100 cells was recorded (c: X-rays; d: protons). At least three independent experiments were performed and data are presented as mean ± 1 SD. Unpaired t-tests were performed (*: $p<0.05$; **: $p<0.01$; ***: $p<0.001$) CTL vs ABT, CTL vs Ola and Ola vs Ola+ABT.

3. The effect of ABT-263 is reduced for cell lines with limited radio-induced senescence

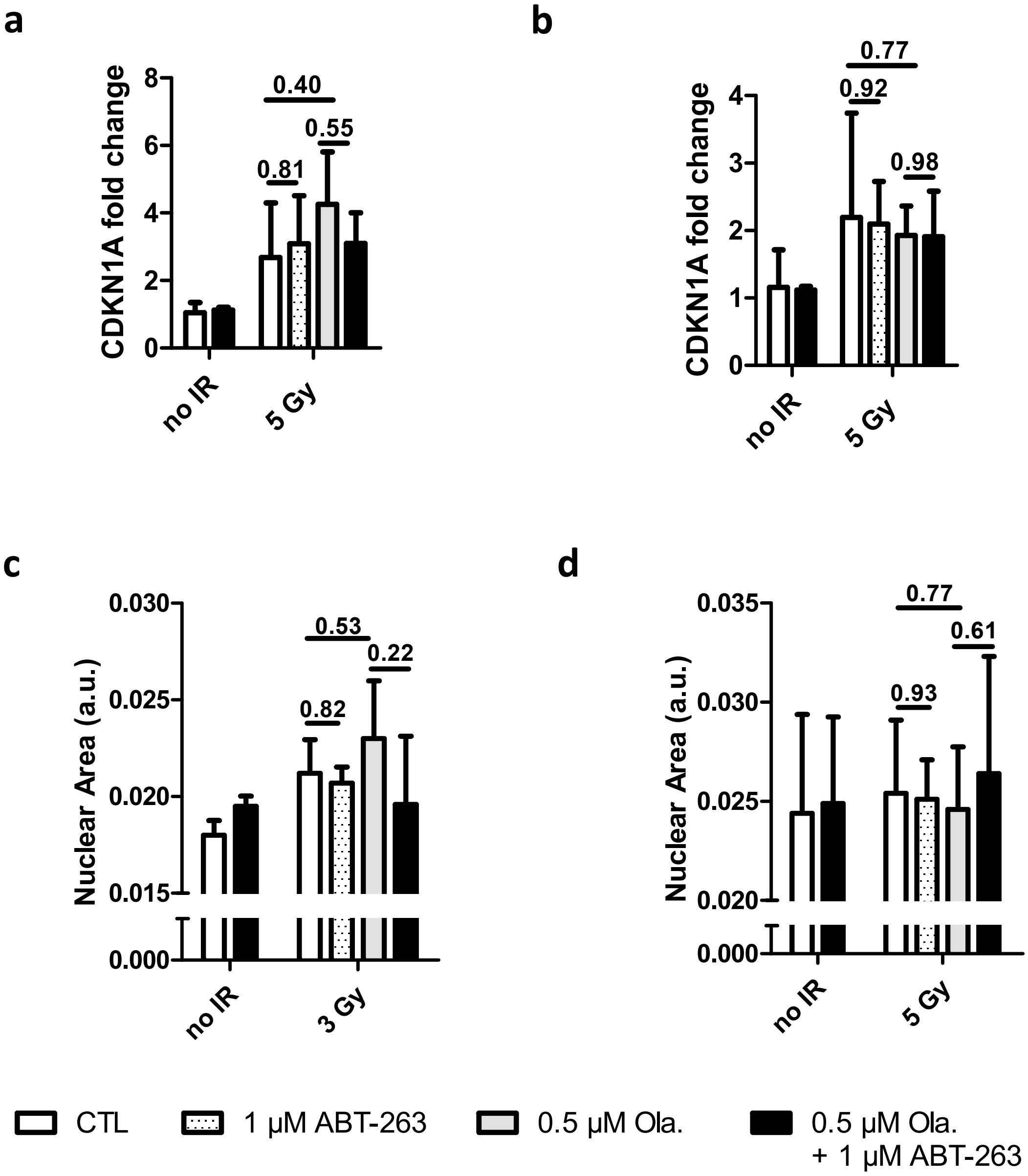

Figure S2: The effect of ABT-263 is reduced for cell lines with limited radio-induced senescence. HCT-116 and KP4 cells were irradiated with 5 Gy X-rays with or without 0.5 µM of Olaparib. 22h after irradiation, the medium was replaced and 1 µM of ABT-263 was added. 6 days after irradiation, mRNA level of CDKN1A was evaluated (a: HCT-116; b: KP4). 6 days post-irradiation, cells were fixed and the nuclei stained with DAPI. The nuclear mean area of at least 100 cells was recorded (c: HCT-116; d: KP4). At least three independent experiments were performed and data are presented as mean ± 1 SD. Unpaired t-tests were performed (*: $p<0.05$; **: $p<0.01$; ***: $p<0.001$) CTL vs ABT, CTL vs Ola and Ola vs Ola+ABT.

4. ABT-263 induces cell death in cell lines displaying radiation-induced senescence

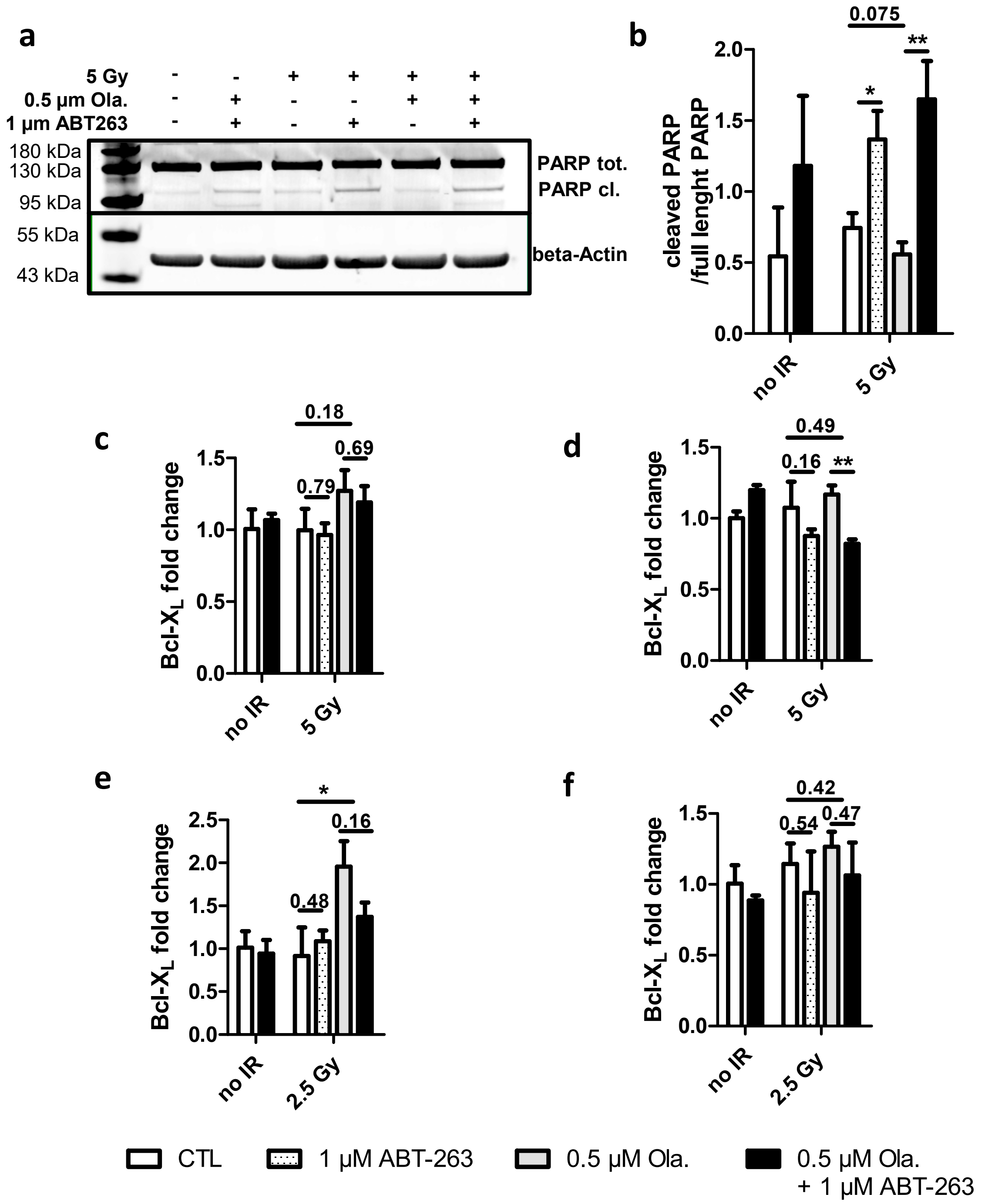

Figure S3: Induction of apoptosis by ABT-263 in A549 cells. A549 cells were irradiated with 5 Gy X-rays with or without 0.5 µM of Olaparib. 22h after irradiation, the medium was replaced and 1 µM of ABT-263 was added. a) Representative western blot results of total and cleaved forms of PARP 6 days after X-ray irradiation. b) Cleaved over full length PARP ratio in A549 cells. At least three independent experiments were performed and data are presented as mean ± 1 SD. c-d) Bcl-XL mRNA level 3 (c) and 6 (d) days after X-ray irradiation. e-f) Bcl-XL mRNA level 3 (e) and 6 (f) days after proton irradiation At least three independent experiments were performed and data are presented as mean ± 1 SD. Unpaired t-tests were performed (*: p<0.05; **: p<0.01; ***: p<0.001) for CTL vs ABT, CTL vs Ola and Ola vs Ola+ABT.

5. **ABT-263 reverts the SASP induced by Olaparib after X-ray and proton irradiation in A549 cells**

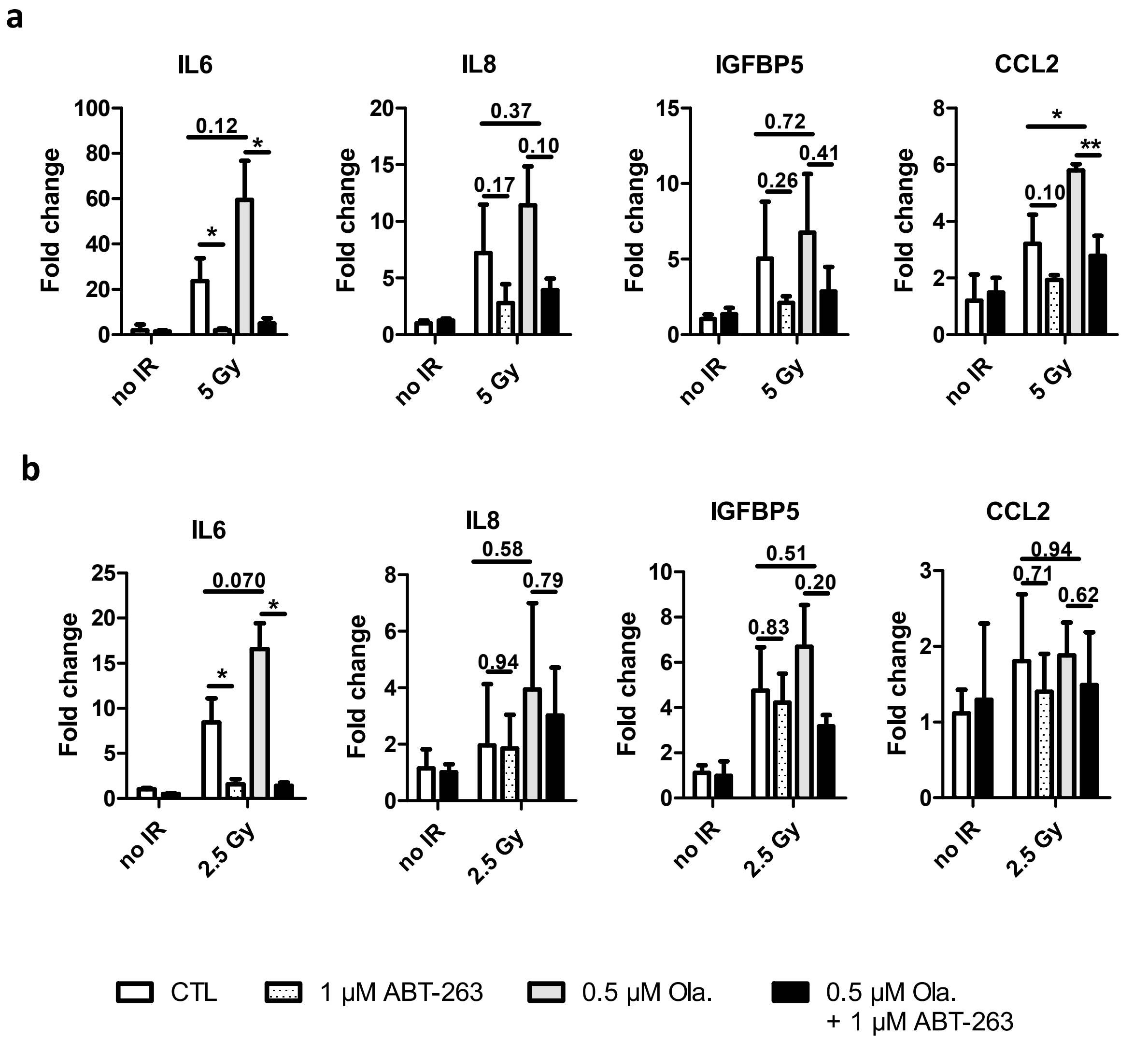

Figure S4: ABT-263 reverts the SASP promoted by Olaparib after X-ray and proton irradiation in A549 cells. A549 cells were irradiated with 5 Gy X-rays or 2.5 Gy protons with or without 0.5 µM of Olaparib. 22h after irradiation, the medium was replaced and 1 µM of ABT-263 was added. a, b) 6 days post-irradiation, mRNA levels of genes implicated in SASP were determined. Fold change, calculated as 2-ΔΔct, are presented after X-rays (a) and protons (b) radiation. At least three independent experiments were performed and data are presented as mean ± 1 SD. Unpaired t-tests were performed (*: p<0.05; **: p<0.01; ***: p<0.001) CTL vs ABT, CTL vs Ola and Ola vs Ola+ABT.

6. X-rays, Olaparib and ABT-263 effects on mRNA level of SASP genes in HCT-116 and KP4 cells

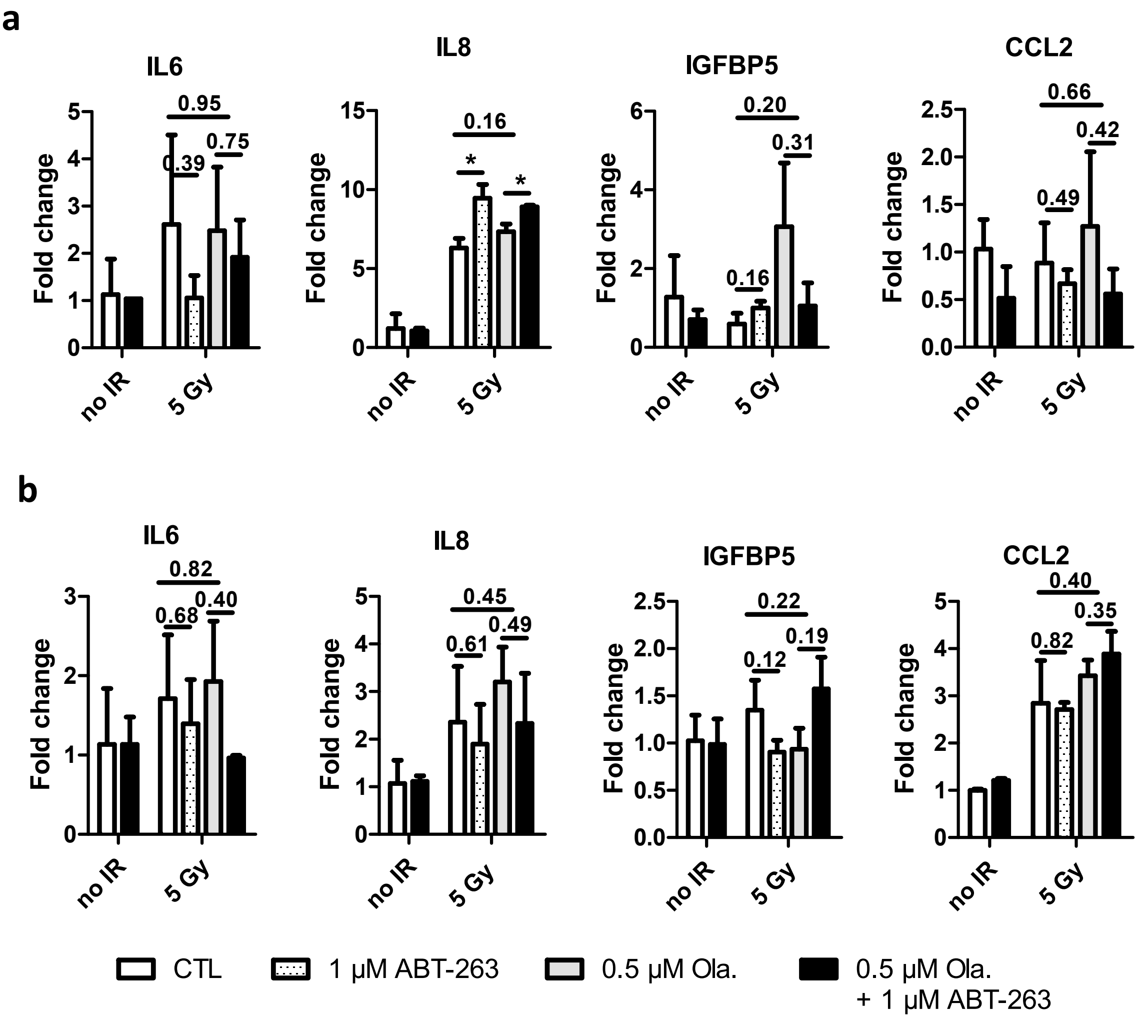

Figure S5: HCT-116 and KP4 cells were irradiated with 5 Gy X-rays with or without 0.5 µM of Olaparib. 22h after irradiation, the medium was replaced and 1 µM of ABT-263 was added. a, b) 6 days post-irradiation, mRNA levels of genes implicated in SASP were determined. Fold change, calculated as 2-ΔΔct, are presented for HCT-116 (a) and KP4 (b) cells. At least three independent experiments were performed and data are presented as mean ± 1 SD. Unpaired t-test were performed (*: p<0.05; **: p<0.01; ***: p<0.001) CTL vs ABT, CTL vs Ola and Ola vs Ola+ABT.

7. Induction of senescence in MDA-MB-231: Effects of Olaparib and ABT-263

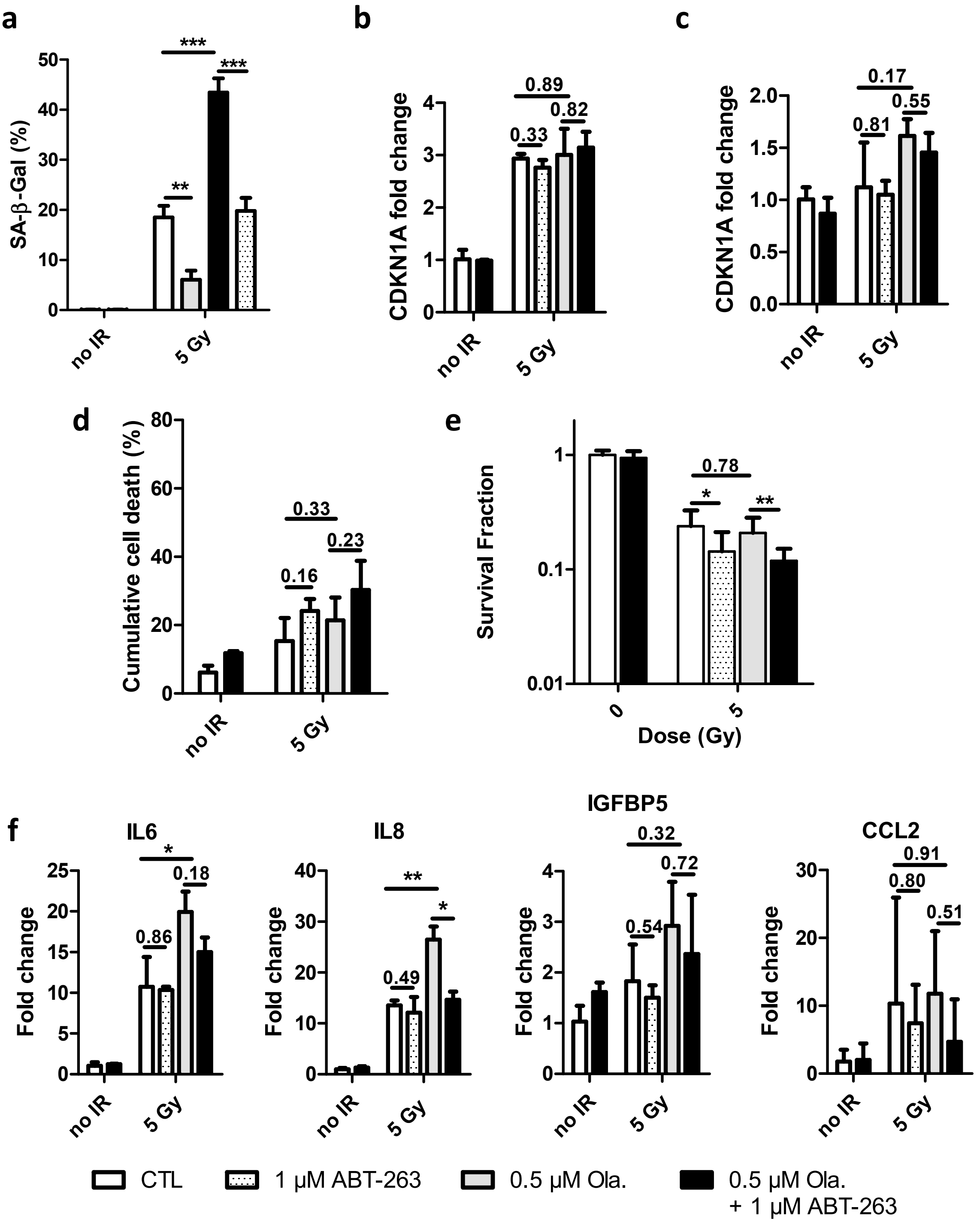

Figure S6: MDA-MB-231 cells were irradiated with 5 Gy X-rays with or without 0.5 µM of Olaparib. 22h after irradiation, the medium was replaced and 1 µM of ABT-263 was added. a) Quantification of SA-β-Gal positive cells 6 days after irradiation. b) CDKN1A mRNA level 3 days after irradiation. c) CDKN1A mRNA level 6 days after irradiation. d) Cumulative cell death 72 h after X-rays. e) Clonogenic survival fraction. F) mRNA levels of genes implicated in SASP were determined 3 days after irradiation. Fold change were calculated as 2-ΔΔct. At least three independent experiments were performed and data are presented as mean ± 1 SD. Unpaired t-tests were performed (*: $p<0.05$; **: $p<0.01$; ***: $p<0.001$) CTL vs ABT, CTL vs Ola and Ola vs Ola+ABT.

8. Correlation between increase in Annexin V and CDKN1A fold change reduction

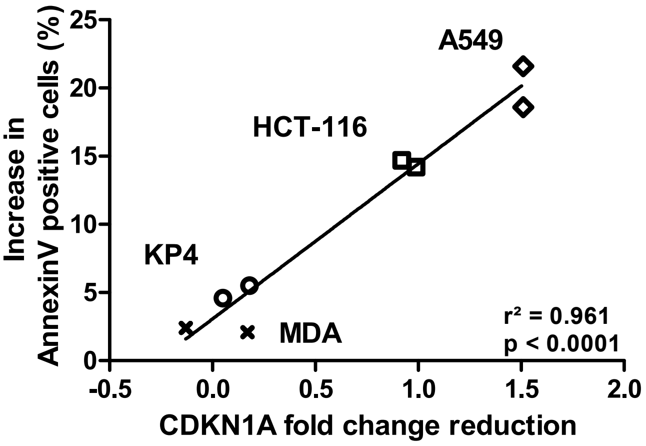

Figure S7: Correlation between the increase in Annexin V and the reduction of CDKN1A fold change at day 3 with the addition of ABT-263. Circle: KP4 cells, square: HCT-116 cells, diamond: A549 cells (X-rays), cross: MDA-MB-231 cells. r^2 and p value obtained with a Pearson's correlation test.

9. Correlation between CDI and reduction of SA-β-Gal by ABT-263

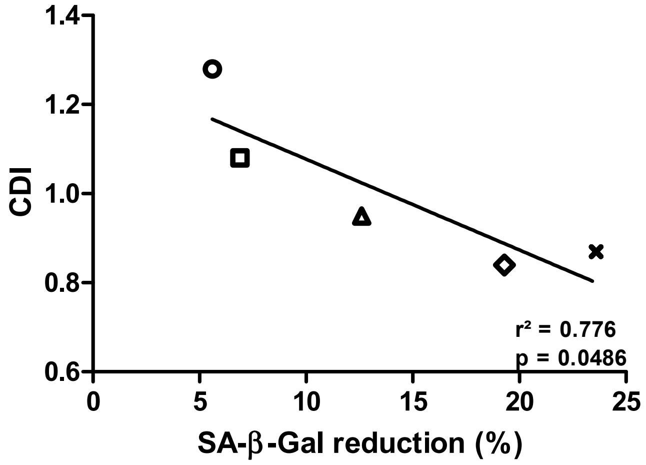

Figure S8: Correlation between CDI and SA-β-Gal activity reduction at 5 Gy with Olaparib with the addition of ABT-263. Circle: KP4 cells, square: HCT-116 cells, triangle: A549 cells (protons), diamond: A549 cells (X-rays), cross: MDA-MB-231 cells. r^2 and p value obtained with a Pearson's correlation test.

REFERENCE

1. Marx, S.; Van Gysel, M.; Breuer, A.; Dal Maso, T.; Michiels, C.; Wouters, J.; Le Calve, B. Potentialization of anticancer agents by identification of new chemosensitizers active under hypoxia. *Biochemical pharmacology* **2019**, *162*, 224-236, doi:10.1016/j.bcp.2019.01.004.
2. Sermeus, A.; Rebucci, M.; Fransolet, M.; Flamant, L.; Desmet, D.; Delaive, E.; Arnould, T.; Michiels, C. Differential effect of hypoxia on etoposide-induced DNA damage response and p53 regulation in different cell types. *Journal of cellular physiology* **2013**, *228*, 2365-2376, doi:10.1002/jcp.24409.
